# Photocatalytic Degradation and Toxicity Analysis of Sulfamethoxazole using TiO_2_/BC

**DOI:** 10.3390/toxics11100818

**Published:** 2023-09-28

**Authors:** Jiatao Dang, Wenjun Pei, Fumeng Hu, Zaihui Yu, Shuheng Zhao, Jianjun Hu, Jiuli Liu, Dongliang Zhang, Zhixuan Jing, Xuan Lei

**Affiliations:** 1 Key Laboratory of New Materials and Facilities for Rural Renewable Energy, Ministry of Agriculture and Rural Affairs, College of Mechanical & Electrical Engineering, Henan Agricultural University, Zhengzhou 450002, China; dangjt1988@163.com (J.D.); zhaosh09@163.com (S.Z.); 2 College of Mechanical and Electrical Engineering, Henan Agricultural University, Zhengzhou 450002, China; pwj1303626895@163.com (W.P.); hfm17550358798@163.com (F.H.); yzh000928@163.com (Z.Y.); l13103668401@163.com (J.L.); zhangdl_369@163.com (D.Z.); jzx2772162035@163.com (Z.J.); 16638938595@163.com (X.L.)

**Keywords:** biochar, photocatalytic degradation, sulfamethoxazole, titanium dioxide

## Abstract

Sulfonamide antibiotics in the environment not only disrupt the ecological balance but can also enter the human or animal body in various forms and cause harm. Therefore, exploring efficient methods to degrade sulfonamide antibiotics is crucial. In this study, we prepared biochar (BC) using corn straw, and TiO_2_/BC was obtained by doping different proportions of TiO_2_ into biochar with varying carbonization temperatures using the sol–gel method. Next, we investigated the degradation of sulfamethoxazole (SMX) in solution using the generated TiO_2_/BC under ultraviolet irradiation and studied the effects of various experimental parameters, such as the type of composite material, composite material addition, solution pH, and initial antibiotic concentration on SMX degradation. Under an initial SMX concentration of 30 mg/L, the composite with the best photocatalytic degradation performance was TiO_2_/BC-5-300 (i.e., 5 mL of TiO_2_ doping; 300 °C calcination temperature), with an addition amount of 0.02 g and a solution pH of 3. The degradation efficiency increased from 22.3% to 89%, and the most significant degradation effect occurred during the initial stage of photocatalytic degradation. In the TiO_2_/BC-5-300 treated SMX solution, the average rhizome length of bean sprouts was significantly higher than that of the untreated SMX solution and slightly lower than that of the deionized aqueous solution (3.05 cm < 3.85 cm < 4.05 cm). This confirmed that the photocatalytic degradation of SMX by the composite was effective and could efficiently reduce its impact on the growth of bean sprouts. This study provides essential data and theoretical support for using TiO_2_/BC in the treatment of antibiotic-contaminated wastewater.

## 1. Introduction

In recent decades, global environmental pollution has become increasingly severe, drawing significant social attention [1]. Among the various types of pollution, water pollution poses a particularly serious threat to human health. Common water pollutants include heavy metals, toxic organic compounds, acid and alkali pollutants, pathogens, and suspended solids. Antibiotics are classified as emerging water pollutants that may lead to ecosystem damage by promoting the transmission of resistance genes, the emergence of drug-resistant bacteria, and other adverse effects. Sulfamethoxazole, a representative sulfonamide antibiotic, is widely used worldwide for the prevention and treatment of bacterial infections in the urethra and other tissues [2]. This compound is water soluble, and its concentration in wastewater from certain croplands can exceed 20 mg/L. Due to the poor biodegradability of antibiotics in wastewater, the traditional activated sludge process used in most sewage treatment plants struggles to effectively degrade antibiotics in sewage. Therefore, there is an urgent need to explore environmentally friendly and effective technologies to remove residual antibiotics from the aquatic environment [3].

To efficiently remove SMX, various advanced technologies have been researched and developed, including adsorption [4], photocatalysis [5], and biodegradation [6]. Photocatalysis is considered one of the most promising technologies for SMX removal due to its low operating cost, high efficiency, and economic viability [7]. TiO_2_ photocatalysts have extensive development potential, being non-toxic, cost-effective, reusable, insoluble in water, and possessing stable chemical properties [8]. However, despite their promising applicability for antibiotic degradation, their low adsorption capacity, high agglomeration, and rapid recombination within a narrow wavelength range (200–400 nm) limit their effectiveness when used alone [9]. As an efficient adsorption material, biochar is often employed to adsorb various heavy metals and organic compounds. The surface of corn straw biochar is rich in functional groups such as hydroxyl, carboxyl, and amino groups, thus facilitating the formation of hydrogen bonds [10] and π–π conjugation [11]. Previous studies have demonstrated that antibiotic residues can be effectively removed using TiO_2_/BC composite materials obtained by loading TiO_2_ onto corn straw biochar by taking advantage of the synergistic effect of the antibiotic adsorption capacities of biochar and the unique properties of TiO_2_ as a catalyzer for pollutant degradation. Relevant studies include Kim et al. [12], who used TiO_2_/BC composites and added bicarbonate during photocatalytic oxidation, thus effectively improving the degradation rate and mineralization of SMX under ultraviolet irradiation. Avramiotis et al. [13] developed a method for oxidizing SMX by activating persulfate with pre-synthesized rice husk biochar (RHB), focusing on key redox activities such as electron transfer, singlet oxygen control, and surface-binding free radicals. Liu et al. [14] demonstrated that a combination of ultraviolet radiation (UV/PDS) can effectively degrade sulfonamide antibiotics. Moreover, using simple solid-phase co-calcination technology, Feng et al. [15] manufactured mesoporous titanium dioxide/chitosan biochar (TiO_2_/CS-BC) that could effectively degrade rhodamine. Kotp [16] used green synthesis technology to prepare Ce-Ti/Cf nanocomposites as photocatalysts to efficiently degrade methylene blue (MB) and methyl orange (MO) dyes. Alahl et al. [17] utilized chitosan/silicate (CHMix) nanocomposites prepared by crosslinking between chitosan and prepared silicate (S2), which can effectively degrade reactive blue and Congo red dyes under sunlight.

In order to explore and obtain efficient methods to degrade sulfonamide antibiotics, we prepared biochar by collecting corn straw, and TiO_2_/BC composites were synthesized using the sol–gel method. Taking SMX as a representative target pollutant, our study analyzed its degradation using TiO_2_/BC under ultraviolet light irradiation, after which we explored the photocatalytic mechanisms of its degradation and conducted toxicological analyses.

## 2. Materials and Methods

### 2.1. Materials

C_16_H_3_O_4_Ti (5593-70-4, AR), C_2_H_6_O (64-17-5, AR), C_2_H_4_O_2_ (64-19-7, AR), HCl (7647-01-0, AR), NaOH (1310-73-2, AR), C_10_H_11_O_3_N_3_S (723-46-6, AR), H_2_O (7732-18-5, AR), and C_6_H_9_N_3_O_2_ (71-00-1, AR).

### 2.2. Preparation of Biochar

In this study, corn straw was used as the raw material for producing biochar. The first step involved crushing the corn straw using a crusher, followed by sieving the crushed material through an 80-mesh sieve to obtain corn straw powder. The obtained powder was then placed in a drying box and dried at 80 °C for 24 h. After drying, the corn straw powder was stored in a sealed bag for later use. Next, a specific amount of corn straw powder was weighed and placed in a tube furnace. The heating rate was set to 10 °C/min, and the final carbonization temperature was set to 500 °C. The corn straw powder was carbonized for 1 h under a protective nitrogen atmosphere. After the carbonization process, the biochar was allowed to cool to room temperature and removed from the furnace.

### 2.3. Preparation of Titanium Dioxide Biochar Composites

**Figure 1:**
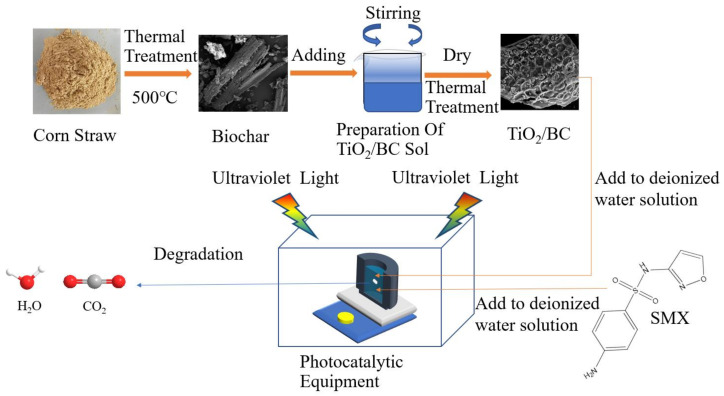
Preparation of TiO_2_/BC composites and photocatalytic degradation flow chart.

TiO_2_/BC composites were prepared using the sol–gel method. Briefly, 20 mL of butyl titanate was added dropwise into bottle A containing 100 mL of absolute ethanol and stirred for 0.5 h. Then, 8 mL of glacial acetic acid, 19.5 mL of distilled water, 50 mL of absolute ethanol, and 0.5 mL of 3.5 mol/L HCl solution were added to bottle B. The contents were stirred for 0.5 h at 40 °C using a magnetic stirrer. Next, the liquid from bottle A was poured into bottle B, and the mixture was stirred for 1 h to form a white gel. The formed wet gel was placed in an evaporation dish, dried at 80 °C, and then ground into a powder. TiO_2_ powder could be obtained by holding at 105 °C temperature for 2 h and calcination at 500 °C for 3 h. The biochar was then weighed (0.01 g) and incorporated into different amounts of the prepared sols (5 mL, 10 mL and 15 mL), after which the mixture was stirred for 30 min. A small amount of distilled water was added to further hydrolyze the mixed gel and reduce its fluidity. The mixture was then dried and ground into a fine powder. Under nitrogen protection, the titanium dioxide biochar composites were prepared at different temperatures (300 °C and 500 °C) using a tube furnace (XD-1200NT, Zhengzhou Brother Kiln Co., Ltd, Zhengzhou, China). The heating rate of the tube furnace was 10 °C/min, and the calcination time was 2 h. The resulting composites were labeled as TiO_2_/BC-A-B, where A represents the doping amount of TiO_2_ (A = 5 mL, 10 mL, 15 mL and 20 mL), and B represents the final calcination temperature of TiO_2_/BC (B = 300 °C and 500 °C). The basic process is shown in Figure 1.

### 2.4. Composite Material Characterization

Several analyses were conducted to gain insights into the structural characteristics of the prepared composites, including scanning electron microscopy (SEM; TESCAN MIRA LMS, TESCAN ORSAY HOLDING, a.s., Brno, Czech Republic), energy dispersive X-ray analysis (EDS; TESCAN MIRA4, TESCAN ORSAY HOLDING, a.s., Brno, Czech Republic), X-ray diffraction analysis (XRD; Panalytical Empyrean, PANalytical B.V. Almelo, The Netherlands), and BET characterization (AUTOSORB IQ, Quantachrome Instrument, Boynton Beach, FL, USA).

### 2.5. Photocatalytic Degradation of SMX

All photocatalytic experiments were conducted using an experimental setup developed in-house consisting of an 8 W UV lamp (A-6090-UVL, Shanghai Chenhong Biotechnology Co., Ltd., Shanghai, China) as a light source and a 200 mL quartz largemouth bottle with a magnetic stirrer (HJ-3, Zibo Runyu Machinery Equipment Co., Ltd., Shandong, China) at a 400 r/min stirring speed. A certain amount of photocatalyst was added to 50 mL of SMX aqueous solution, after which the photocatalytic degradation experiment was continuously performed for 4 h under ultraviolet irradiation. The pH of the solution was adjusted using 0.1 mol/L hydrochloric acid and sodium hydroxide solutions at room temperature. At predetermined intervals, solution samples were extracted from the suspension and filtered using a 0.45 μm filter. The photocatalytic degradation of SMX was evaluated using a UV-Vis spectrophotometer (U-3900 type, Hitachi Hi-Tech Science Naka Works, Japan Co., Ltd, Hitachi-Naka City, Ibaraki Prefecture, Japan) at a maximum wavelength (λ) of 283 nm.

### 2.6. Adsorption Experiment

The influence of different materials on SMX adsorption may be different. According to the preparation method of Section 2.2 and Section 2.3, different adsorbent materials were prepared, respectively recorded as BC, TiO_2_, and TiO_2_/BC-5-300. A total of 20 mg of different materials were weighed into Erlenmeyer flasks, which measured 50 mL of 20 mg/L SMX solution. An amount of 0.1 mol/L hydrochloric acid and 0.1 mol/L sodium hydroxide were used to adjust the pH of the solution to neutral conditions. The solution was stirred magnetically for 4 h at room temperature, with a speed of 400 r/min. The supernatant was filtered through 0.45 μm membrane at 60 min, 120 min, 180 min, and 240 min, respectively. The absorbance of the remaining SMX in the solution was determined at wavelength of 283 nm. The equilibrium adsorption capacity was calculated, and the adsorption kinetic curve was drawn.

### 2.7. Stability and Reusability

TiO_2_/BC-5-300 was prepared according to the preparation method of Section 2.3. A total of 20 mg TiO_2_/BC-5-300 was weighed into an Erlenmeyer flask, and 50 mL of 20 mg/L SMX solution was measured in each Erlenmeyer flask. The pH of the solution was adjusted to neutral conditions with 0.1 mol/L hydrochloric acid and 0.1 mol/L sodium hydroxide. The solution was stirred magnetically for 4 h at room temperature, with a speed of 400 r/min. The supernatant was filtered through 0.45 μm membrane. The absorbance of the remaining SMX in the solution was determined at wavelength of 283 nm. After sampling, the solution was repeatedly rinsed with deionized water and dried to obtain the photocatalytic material until the experiment was repeated three times.

## 3. Results and Discussion

### 3.1. Characterization Analysis of Composite Material

#### 3.1.1. XRD Analysis

**Figure 2:**
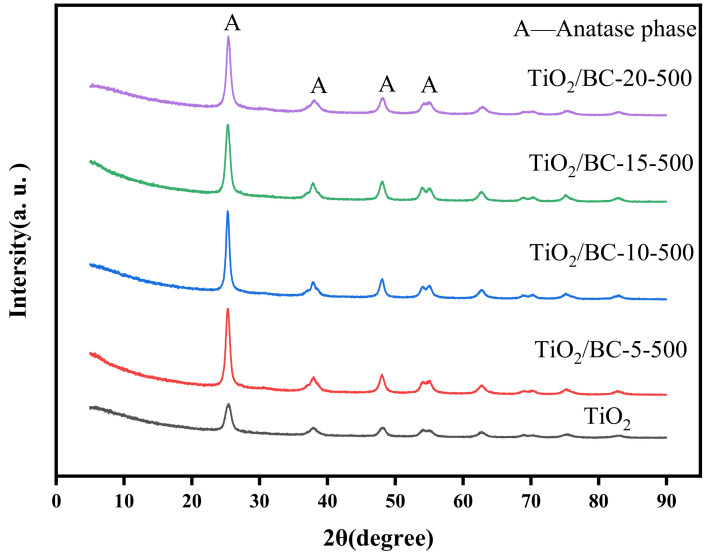
X-ray diffraction (XRD) patterns of TiO_2,_ TiO_2/_BC-5-500, TiO_2_/BC-10-500, TiO_2_/BC-15-500, and TiO_2_/BC-20-500.

In Figure 2, the main characteristic peaks in the XRD pattern of sample TiO_2_/BC-500 indicate that it mainly consists of anatase in crystal form (JCPDS 21-1272) [18]. The prepared TiO_2_ nanoparticles exhibit an amorphous structure, with peaks observed at 2θ values of 15.33°, 37.8°, 48.1°, 53.9°, 55°, 62.18°, and 68.8°, corresponding to the (101), (004), (200), (105), (211), (213), and (116) planar configurations, respectively. These peaks are well matched with the standard card (JCPDS NO. 84-1286) [19], indicating the successful preparation of titanium dioxide nanoparticles. For biochar, diffraction peaks were observed at 2θ = 26.6° and 2θ = 45°, corresponding to the carbon structure’s (002) and (100) planes (JCPDS 01-0640) [20]. The diffraction peak of biochar at 2θ = 26.6° is similar to the diffraction peak of anatase TiO_2_ at 2θ = 25.3°. It is calculated that the FWHM of TiO_2_, TiO_2_/BC-5-500, TiO_2_/BC-10-500, TiO_2/_BC-15-500, and TiO_2_/BC-20-500 are 1.0033, 0.7682, 0.6656, 0.8283, and 0.8075, while the d-spacing are 0.1572, 0.2055, 0.2372, 0.1906, 0.1956, and 0.1956, respectively [21]. However, after calcination with different doping ratios, the diffraction peak of biochar in the sample does not appear, indicating the successful incorporation of biochar and TiO_2_ doping. The diffraction peak pattern of the TiO_2_/BC composites is generally consistent with that of pure TiO_2_, indicating good dispersion of titanium dioxide nanoparticles in the biochar matrix. Moreover, the preparation process of TiO_2_/BC composites does not affect the crystal form of TiO_2_, and anatase was the dominant crystal form of the composite.

#### 3.1.2. SEM-EDS Analysis

**Figure 3:**
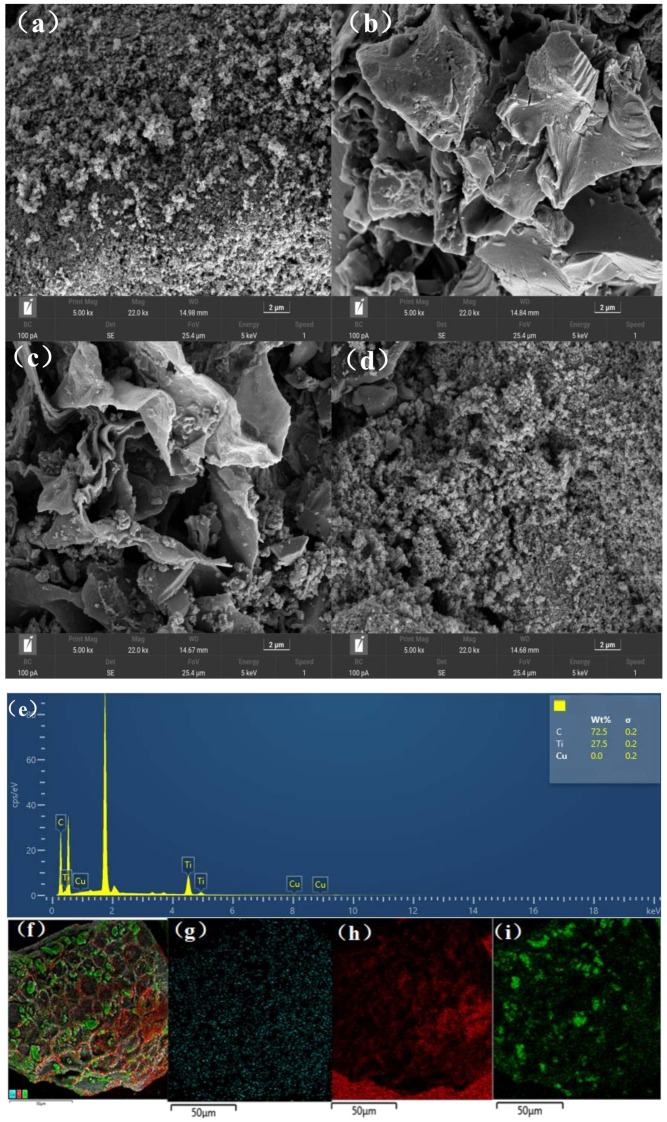
TiO_2_ (**a**), TiO_2_/BC-5-500 (**b**), TiO_2_/BC-10-500 (**c**), and TiO_2_/BC-15-500. (**d**) SEM diagram and TiO_2_/BC-5-500 EDS spectra (**e**) and image (**f**). TiO_2_/BC-5-500 EDS element mapping images of Cu (**g**), C (**h**), and Ti (**i**).

SEM and EDS are effective analysis techniques that can provide valuable insights into the topography of TiO_2_/BC, as well as the TiO_2_ loading information on BC, respectively. As illustrated in Figure 3a, TiO_2_ occurred as both small particles and large non-homogeneous particle structures, with some particles agglomerated together. After carbonization at 500 °C for 1 h under N_2_ gas shielding, the biochar exhibited a rich porous structure. As illustrated in Figure 3b,c, the doping of TiO_2_ and BC causes TiO_2_ to attach to BC, forming a coral-like structure without significant pore blockage. The small pores in the composite greatly increase its surface area, thus facilitating the capture of SMX and light energy and improving the catalyst’s efficiency. Figure 3d shows numerous pore structures, irregular surfaces, and mesoporous structures similar to wormholes, which further enhance TiO_2_ attachment and achieve better adsorption effects.

The EDS analysis results in Figure 3f reveal the presence of many microporous structures inside the carbon material, providing a favorable surface for titanium (Ti) adhesion. Titanium exists in two main forms: one with convex surfaces and long bands attached to the biochar skeleton and the other appearing as blocky structures partially attached to the biochar. TiO_2_ is uniformly distributed within the carbon material, with an approximately 3:1 C-to-Ti ratio. Combined with the results of our XRD pattern analysis, our findings confirmed that elemental Ti existed as TiO_2_ in the anatase crystal form. The presence of carbon facilitates light absorption and promotes the separation of photoelectrons. Moreover, the addition of carbon helps the TiO_2_/biochar composite to inhibit the recombination of holes and electrons [22], which aids in the degradation of SMX by TiO_2_.

#### 3.1.3. Pore Size Analysis of Composite Materials

The specific surface area and pore structure characteristics of TiO_2_/BC-500 and BC-500 were determined through low-temperature N_2_ adsorption–desorption characterization, and the calculated specific surface area and associated pore volume are presented in Table 1. The specific surface area of TiO_2_/BC composites was found to be smaller than that of pure BC particles. The specific surface area of BC was measured to be 63.435 m^2^/g, falling within the expected range for biochar. However, for TiO_2_/BC-5-500, the BET surface area decreased by 26.709 m^2^/g. This reduction can be attributed to the attachment of TiO_2_ onto the surface of the biochar, covering part of the mesopores and micropores, consistent with previous studies [23]. Despite this decrease in specific surface area, TiO_2_/BC-5-500 exhibited significant beneficial synergistic advantages compared to BC-500. The support structure provided by TiO_2_ made the surface active site distribution of the composites more uniform, resulting in a higher average pore size compared to BC-500. Moreover, this modification increased the content of oxygen-containing functional groups in the composite, thus promoting the diffusion of organic pollutants to the surface of TiO_2_/BC-5-500 composites for photocatalytic degradation.

**Figure 4:**
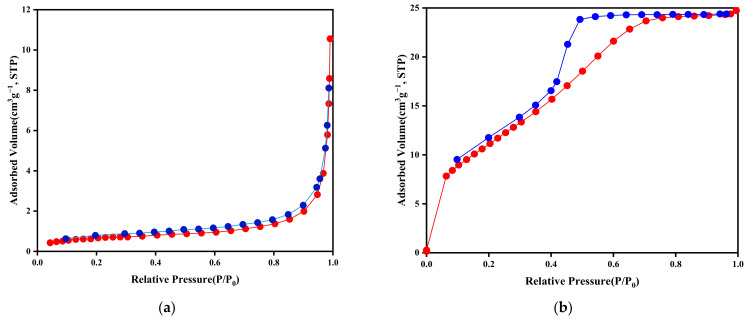
N_2_ adsorption and desorption curve of BC-500 (**a**) and TiO_2_/BC-5-500 (**b**).

Figure 4 illustrates the N_2_ adsorption–desorption curves of BC-500 and TiO_2_/BC-5-500. The red line represents the nitrogen adsorption curve, and the blue line represents the nitrogen desorption curve.The N_2_ adsorption–desorption curve of BC-500 follows a typical type (III) curve, indicating the dominance of a microporous structure in the original BC-500. On the other hand, the N_2_ adsorption–desorption curve of TiO_2_/BC-5-500 exhibits a type (IV) curve with a hysteresis loop, suggesting that TiO_2_/BC-5-500 is primarily composed of a mesoporous structure [24]. The pore volume of TiO_2_/BC-5-500 is 17.7 times that of BC-500, indicating a transformation of the pore structure from microporous to mesoporous after TiO_2_ modification. This change is conducive to enhancing the ability of TiO_2_/BC-5-500 to adsorb SMX, making it more effective for photocatalytic degradation.

### 3.2. Photocatalytic Degradation of Antibiotic Experiments

#### 3.2.1. Photocatalytic Degradation Effect of Different Catalysts on SMX

**Figure 5:**
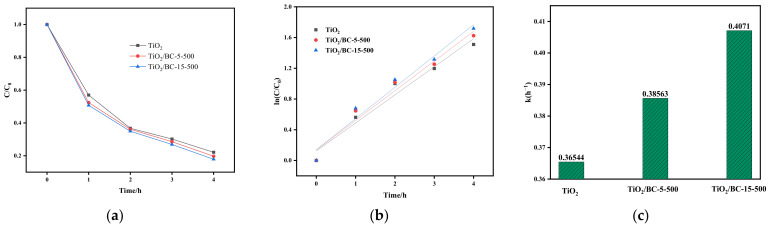
(**a**) Comparison of degradation of SMX by different composites (catalyst concentration: 0.02 g/L, SMX concentration: 20 mg/L, and pH: 3). (**b**,**c**) Pseudo-primary reaction kinetics and kinetic constants of SMX degradation corresponding to the prepared sample.

TiO_2_/BC composites exhibit unique structural, morphological, and compositional advantages, making them suitable as both adsorption and photocatalytic materials [25]. As shown in Figure 5a, under ultraviolet irradiation, the addition of TiO_2_/BC as a photocatalytic material increases the removal efficiency of SMX by 7% compared to using single titanium dioxide particles. This indicates that the photocatalytic activity of TiO_2_/BC composites is higher than that of individual titanium dioxide particles. Furthermore, the removal rate of SMX increases with the content of titanium dioxide in TiO_2_/BC composites compared to using titanium dioxide particles alone. The degradation rate of SMX by each catalyst tended to increase first and then decrease as the reaction progressed. Among the different TiO_2_/BC composites, the TiO_2_/BC-15-500 system exhibits the highest degradation efficiency. The photocatalytic degradation kinetic curve of SMX was fitted with the pseudo-first-order kinetic equation, which successfully describes the degradation process [26].

ln(C/C_0_) = kt (1)

where C (mg/L) is the concentration of SMX in solution, C_0_ (mg/L) is the initial concentration of SMX, k (h^−1^) is the reaction rate constant, and t is the duration of the photocatalytic reaction. All data are in full agreement with the pseudo-first-order kinetic curve. The corresponding k value is shown in Figure 5c. Notably, the k value of the TiO_2_/BC-15-500 system (0.4071 h^−1^) is 1.115 times and 1.055 times that of TiO_2_ (0.36544 h^−1^) and TiO_2_/BC-5-500 (0.38563 h^−1^), respectively, indicating that higher proportions of titanium dioxide in the composite led to a significant increase in the surface-active sites in the composite, resulting in an enhanced photocatalytic ability for SMX degradation.

#### 3.2.2. Adsorption Experiments

**Figure 6:**
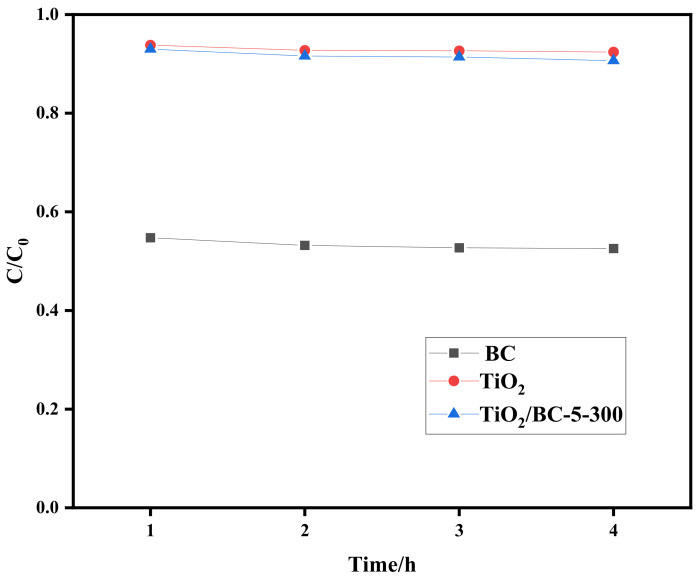
Study of adsorption performance of (BC, TiO_2_, and TiO_2_/BC-300).

The adsorption performance of prepared BC, TiO_2_, and TiO_2_/BC-300 on SMX was investigated by adsorption experiments. The content of SMX in the solution before and after adsorption was determined by an ultraviolet–visible spectrophotometer at λ = 283 nm. The change in SMX concentration was expressed by C/C_0_, C (mg/L) is the remaining concentration of SMX in the solution after adsorption equilibrium, and C_0_ (mg/L) is the SMX concentration in pre-adsorption solution. It can be seen from Figure 6 that the adsorption performance of TiO_2_ and TiO_2_/BC-5-300 for antibiotic SMX is not ideal, and the removal rate of SMX is 7.6% and 9.35%. Compared with TiO_2_ and TiO_2_/BC-5-300 materials, the BC removal rate of SMX is improved to a certain extent, with a removal rate of 47.45%, which is more than six times higher than that of TiO_2_ and TiO_2_/BC-5-300 materials.

#### 3.2.3. Effects of Different Initial SMX Concentrations, Solution pH, and Composite Material Addition on SMX Removal

**Figure 7:**
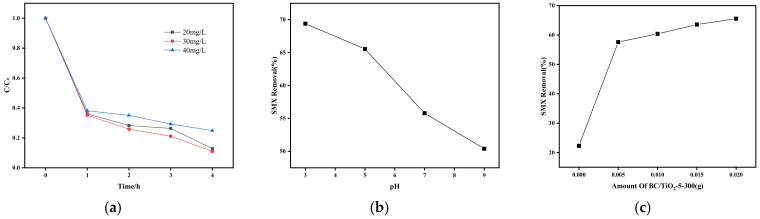
(**a**) Comparison of degradation curves of different initial concentrations of SMX (catalyst concentration: 0.02 g/L, catalyst type: TiO_2_/BC-5-300, and pH: 3). (**b**) Effect of pH value of the solution on the degradation of SMX (catalyst concentration: 0.02 g/L, catalyst type: TiO_2_/BC-5-300, and initial concentration of SMX: 20 mg/L). (**c**) Effect of catalyst usage on the degradation of SMX (SMX concentration: 20 mg/L, catalyst type: TiO_2_/BC-5-300, and pH: 5).

As depicted in Figure 7a, the photocatalytic degradation reaction of SMX is primarily concentrated in the first hour of the initial reaction, during which the degradation rate is the fastest and can reach over 60%. This indicates that the degradation of antibiotics in the photocatalytic reaction process is relatively rapid in the initial stage, after which the degradation rate gradually decreases over time. This decline in degradation rate may be due to the availability of a large number of unoccupied surface sites for photocatalytic degradation in the initial stage of the reaction. As the reaction progresses, the amount of SMX degradation decreases due to the saturation of SMX mineralized on the surface of TiO_2_/BC-5-300. Among the tested initial concentrations of SMX, the fastest degradation rate was observed at 30 mg/L, with a degradation rate of 89%. However, as the initial concentration of SMX increased from 30 mg/L to 40 mg/L, the degradation rate decreased from 89% to 75%. This suggests that the degradation rate of SMX decreases with increasing SMX concentrations under a certain amount of photocatalyst. The reason for this phenomenon is that the number of active groups and electron–hole pairs remains fixed when the same dose of photocatalyst is added. At low concentrations of SMX, there are sufficient active radicals to efficiently break down and mineralize each SMX molecule into smaller molecules. On the other hand, at high SMX concentrations, the existing reactive radicals are not sufficient to mineralize the excess SMX molecules. Additionally, more intermediates are produced as the SMX molecules are degraded. In turn, these intermediates can cover the surface of the photocatalyst, thus inhibiting the absorption of visible light and the generation of electrons and holes, leading to a decrease in the degradation rate.

The pH of the SMX solution is a critical parameter that significantly affects the SMX removal behavior and possible removal mechanism of TiO_2_/BC-5-300 composites. Therefore, the SMX removal efficiency of the composites was examined at pH 3, 5, 7, and 9. The results shown in Figure 7b demonstrate that as the pH of the solution increases, the removal rate of SMX decreases. At a pH of 3, the degradation rate of SMX is relatively high, reaching 69.4%. However, as the pH value of the solution increases to 5, the degradation rate of SMX decreases slightly. When the pH value of the solution is 7 or 9, the removal rate of SMX is significantly reduced, with a removal rate of 50.4% at pH 9. Therefore, the photocatalytic degradation rate of TiO_2_/BC-5-300 composite on SMX is better in acidic environments compared to neutral and weak alkaline conditions. This suggests that the pH value of the SMX solution is closely related to the photocatalytic degradation rate. As the pH value increases, the surface properties and surface charge of the TiO_2_/BC composites change, leading to a significant decrease in the photocatalytic degradation rate. The TiO_2_/BC composite maintains a cationic form when the pH of the solution is less than 1.7 and an anionic form when the pH is greater than 5.6 [27]. With a pKa value of 6.1, the biochar titanium dioxide composite remains positively charged when the pH of the solution is below 6.1 and negatively charged when it is above 6.1. Therefore, under acidic pH conditions (pH = 4), the surface of the titanium dioxide/biochar composite maintains a positive charge, which may electrostatically attract the negatively charged structures on the SMX surface for adsorption. Once adsorbed on the TiO_2_/BC surface, SMX can be degraded by the OH radicals generated by the photocatalysis of TiO_2_/BC composites.

As illustrated in Figure 7c, the SMX removal effect of four different concentrations (0.005, 0.010, 0.015, and 0.02 g/L) of TiO_2_/BC-5-300 composites was also studied. As the amount of TiO_2_/BC-5-300 composites increases from 0 to 0.005 g/L and 0.02 g/L, the degradation efficiency of SMX also increases from 22.3% to 57.6% and 65.55%. This indicates that with the increased addition of TiO_2_/BC-5-300 composites, more active sites and electron holes are generated for the mineralization of SMX, resulting in a more thorough degradation of SMX. Among the concentrations examined herein, the catalyst exhibited the best catalytic effect when 0.02 g of it was added.

#### 3.2.4. Analysis of Photocatalytic Degradation Mechanism of SMX by TiO_2_/BC

**Figure 8:**
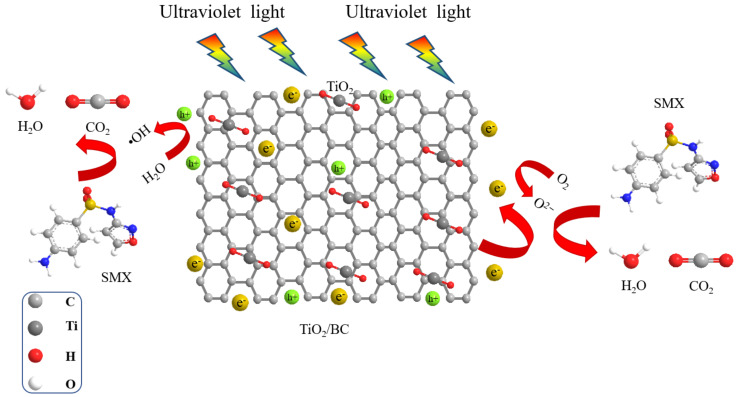
Diagram of the mechanism of photocatalytic degradation of SMX by TiO_2_/BC.

Biochar, as a supporting material compounded with TiO_2_, contributes to the photocatalytic degradation of SMX for two main reasons. Firstly, it provides an adsorption mesh with a porous surface, enhancing the adsorption capacity of SMX molecules. Secondly, the formation of composite materials can prevent the recombination of electrons (e^−^) and holes (h^+^) and produce a large number of π electrons, thereby reducing the band gap and improving photocatalytic efficiency [28]. Moreover, when BC and TiO_2_ photocatalytic composites are exposed to high photon energy incidence, they generate h^+^ and e^−^ pairs that are excited to the valence bands. The photogenerated h^+^ can freely migrate to the surface of titanium dioxide, where it reacts with water to produce highly reactive OH radicals [29]. Similarly, the photogenerated e^−^ in the titanium dioxide conduction band reacts with O_2_ to form O^2−^ [30]. The detailed reaction for the generation of OH and O^2−^ is shown in Figure 8. Both of these free radicals, OH and O^2−^, have a strong ability to oxidize SMX and its intermediates to carbon dioxide, water, etc. [31]. The chemical reactions for the photocatalytic formation of OH and O^2−^ are as follows:

SMX+ (OH, O^2−^) → products (2)

To determine the type of intermediately generated active species, a free radical scavenger can be added to the TiO_2_/BC system for classical quenching experiments. O^2−^ can be quenched by L-histidine (LH) [32]. When the concentration of L-H in the reaction system is 0.01 g, only a degradation rate of 13.1% can be achieved. Compared with the addition of L-H, the degradation efficiency of SMX was reduced by 80.5%, and the above data showed that O^2−^ was generated in the TiO_2_/BC reaction system and participated in the reaction, and O^2−^ contributed greatly.

### 3.3. Analysis of SMX Toxicity

**Figure 9:**
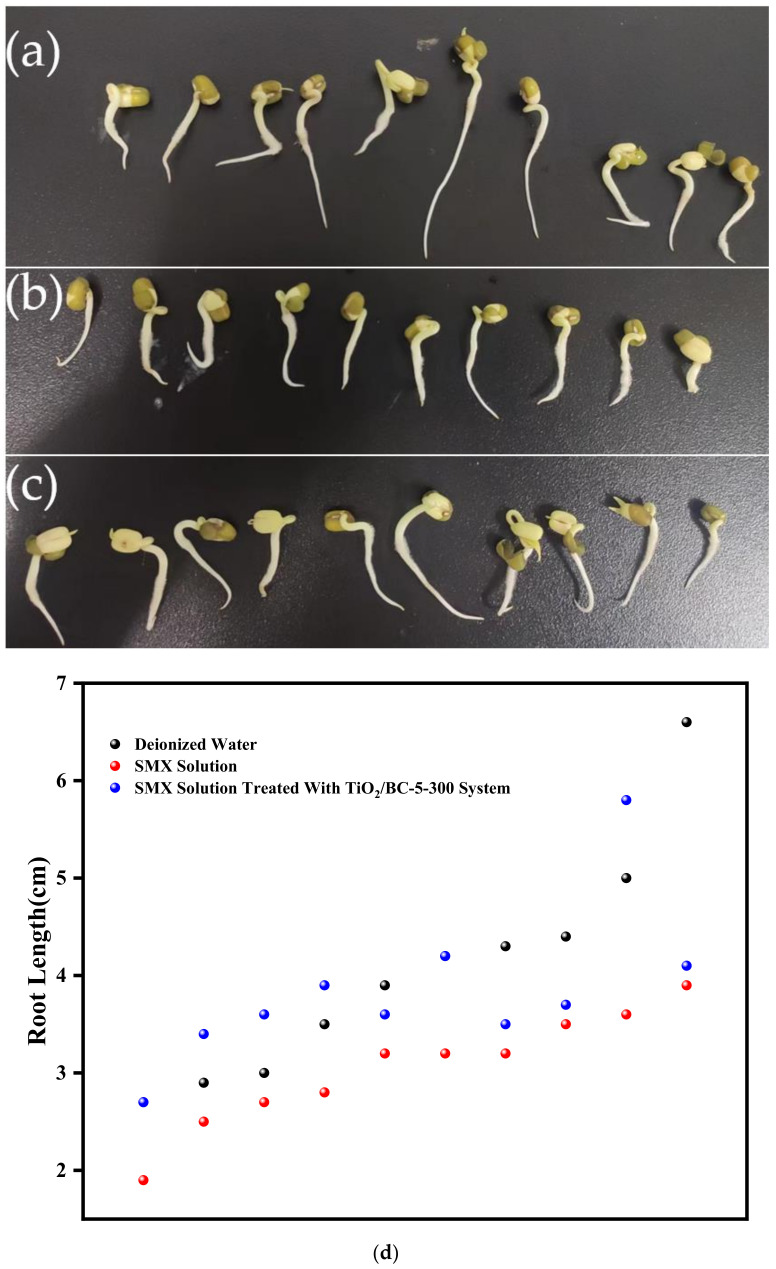
Growth of mung bean sprouts after culture in (**a**) deionized water, (**b**) SMX solution, and (**c**) SMX solution after TiO_2_/BC-5-300 treatment. (**d**) Scatter plot of the length of the rhizome of bean sprouts.

Mung bean sprouts were cultured using the TiO_2_/BC-5-300 photocatalytically treated SMX solution to assess the biological toxicity of the final reaction solution. Mung bean seeds were soaked at room temperature for 4 h and then incubated at 35 °C for 2 days in Petri dishes containing equal amounts of deionized water, SMX solution, and TiO_2_/BC-5-300-treated SMX solution. After treatment with deionized water, SMX solution, and treated SMX solution, the average length of mung bean sprout roots was measured to be approximately 4.05 cm, 3.05 cm, and 3.85 cm, respectively (Figure 9d). In the TiO_2_/BC-5-300 treated SMX solution, the bean sprouts exhibited significantly higher growth rates than in the untreated SMX solution but slightly lower growth rates than in the deionized aqueous solution. These results indicate that after TiO_2_/BC-5-300 treatment, the SMX in the solution was effectively broken down, and the toxicity of SMX to plants was significantly reduced compared to the untreated SMX solution. This suggests that the photocatalytic degradation process using TiO_2_/BC-5-300 was successful in reducing the harmful effects of SMX on plant growth.

### 3.4. Stability and Reusability

**Figure 10:**
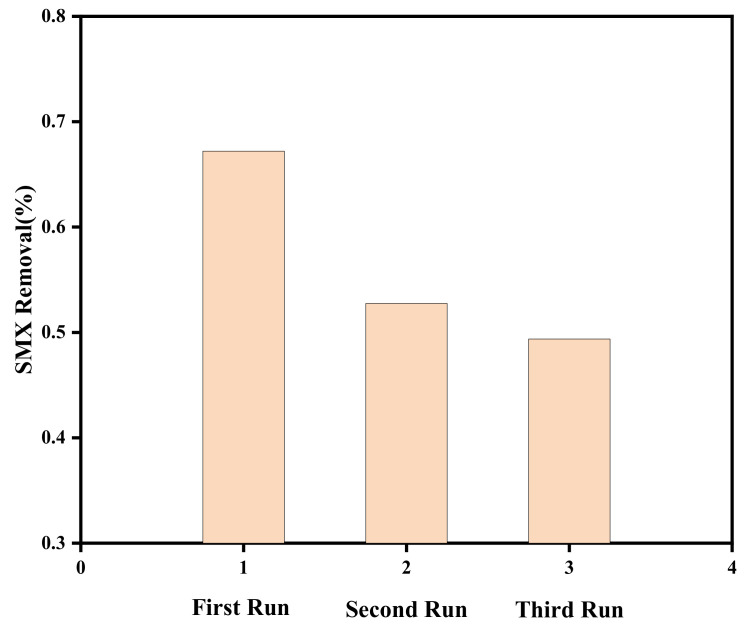
Three-cycle experiment of TiO_2_/BC-5-300 material degradation of SMX.

Good stability and repeatability can reduce application costs. In Figure 10, it can be seen that TiO_2_/BC-5-300 can degrade SMX with a efficiency of 49.38% after three repeated operations,, indicating that the synthesized catalyst shows good reusability.

## 4. Conclusions

In this study, corn straw was chosen as the raw material for producing biochar. TiO_2_ sol prepared using the sol–gel method and doped with different molar ratios of titanium dioxide was subjected to different calcination temperatures to obtain TiO_2_/BC composite catalytic materials. The TiO_2_/BC composite catalytic materials were thoroughly characterized and analyzed. Afterward, the photocatalytic degradation effect of the prepared composites on SMX was examined. After examining the toxicity of SMX solution to plants after treatment with the composite, the following conclusions were obtained:

The specific surface area of TiO_2_/BC composites is smaller compared to that of pure BC particles. However, the addition of biochar promotes the utilization of ultraviolet light by the composites and effectively inhibits the recombination of photogenerated electrons and holes. Notably, at a calcination temperature of 500 °C, the composite materials exhibited improved crystallization, with the main mineral type being the anatase phase. The pore volume of TiO_2_/BC-5-500 was 17.7 times larger than that of BC-500, thus significantly enhancing the adsorption capacity of TiO_2_/BC-5-500 for SMX. This improved adsorption capability makes SMX more susceptible to photocatalytic degradation.

Under our study conditions, the degradation rate of SMX for each catalyst tended to increase first and then decrease as the reaction progressed, and all data were fully consistent with the pseudo-first-order kinetic curve. The photocatalytic degradation reaction was primarily concentrated within the first hour of the initial reaction, with SMX removal exceeding 60%. The carbon composite material showing the most effective degradation of SMX solution was TiO_2_/BC-5-300, achieving a degradation rate of 89% when 0.02 g of the composite was added and the reaction was maintained at pH 3.

Mung bean sprouts cultured using the TiO_2_/BC-5-300 treated SMX solution exhibited higher growth rates (average root length 3.85 cm) than mung bean sprouts cultured in untreated SMX solution (3.05 cm) but slightly lower growth rates than those cultured in deionized aqueous solution (4.05 cm). These findings demonstrate that the TiO_2_/BC photocatalytic system could effectively reduce the toxicity of the SMX solution.

## Figures and Tables

**Table 1:** The BET value of BC-500 and TiO_2_/BC-5-500.

Material	BET Surface Area (m^2^/g)	BJH Adsorption Volume Pores (cm^3^/g)	Adsorption Mean Pore Diameter (nm)
BC-500	63.435	0.5573	25.967
TiO_2_/BC-5-500	36.726	9.8499	3.5567

## Data Availability

Not applicable.
